# Pain and (e)motion in postural responses

**DOI:** 10.3389/fnhum.2013.00286

**Published:** 2013-06-19

**Authors:** Stefano Sandrone, Adam Safron

**Affiliations:** ^1^NATBRAINLAB - Neuroanatomy and Tractography Brain Laboratory, Department of Forensic and Neurodevelopmental Sciences, Institute of Psychiatry, King's CollegeLondon, UK; ^2^Institute of Neuroinformatics, University of Zurich and ETH ZurichZurich, Switzerland; ^3^Department of Psychology, Northwestern UniversityEvanston, IL, USA

Unfortunately, we all know the unpleasant sensory and emotional experience of pain. What we can now explore scientifically in regards to pain has been complexly expressed through language and philosophy. The ancient Greeks used the word *pathos*, originally meaning “something that happens, even despite our willingness,” and which soon shifted to have broader applicability. The Athenian statesman and poet Solon (638–558 BC) believed that “no man can be happy,” being “wretched all those who contemplate the sun.” One century later, the lyric poet Pindar (c. 522–443 BC) wrote that there has never been a man “devoid of trouble” on earth, and there will never be one. In a recent *Frontiers* article, inspired by up-to date neuroscience evidence on the perception of pain, Lelard and colleagues investigated the interrelations between behavior and emotion in painful situations (Lelard et al., 2013).

To determine how imagining oneself in painful situations might influence muscular and physiological responses, posturographic and physiological data were collected from participants viewing pictures of both painful and non-painful situations, while instructed to imagine themselves experiencing each situation. A displacement of center-of-pressure measure collected from a posturographic platform indicated that movement in the anteroposterior direction was shorter when subjects were presented with painful images. Responses of the tibialis anterior and soleus muscles, which have been associated with freezing responses in previous reports (Carpenter et al., 2001), were also collected, and electrocardiographic and electrodermal activities were recorded. The authors showed that higher freezing activity was associated with painful images, although variability of tibialis anterior and root mean square soleus muscle values did not differ significantly between painful and non-painful situations. Interestingly, electrodermal activity and heart rate did not significantly differ between types of image stimuli, indicating that posturographic measures may have greater sensitivity in some contexts. Results thus supported the authors' hypothesis that active attempts to embody displays of painful situations would induce measurable postural adaptation variations.

Previous investigations have differentiated the “representational characters” that emerge while imagining a situation from the “instinctual characters” that arise in more automatic bodily responses, and have suggested that these may emerge from cortical vs. subcortical activity, respectively (Giummarra et al., 2008; Michalak et al., 2009; Kiefer and Pulvermuller, 2012). Although the “instinctual” nature of visceral or subcortically-driven responses is unclear, it is reasonable to distinguish these sorts of reactions from cognitive and affective processes relying on deliberative imagining. The present study sought to examine whether responses aligned with more representational-type processes, ones that would be presumably more strongly involved in perspective-taking would evoke postural responses similar to those resulting from images evoking more visceral responses.

The article by Lelard and colleagues suggests that emotional content is capable of changing muscular responses in specific configurations with ecological significance (Lelard et al., 2013). That is, contemplating painful situations may result in patterns of muscle activity that correspond to freezing responses. A question of particular interest in considering how content might interact with physiological response is the extent to which “representational” tasks involve automatic, visceral responses, as well as the extent to which automatic responses involve (perhaps learned) representation. To what extent can these responses be mediated by innate brainstem or spinal-column reflexes? How closely are they mediated by conditioned associations from a lifetime of experience? To address such questions, future research should explore physiological responses to stimuli differentiated by level of representation and visceral responses evoked. Ideally, these questions would be examined through cross-sectional designs to examine responses at different ages with different extents of learning experiences.

Additional questions may inform future research. For example, in what situations might emotionally-laden stimuli evoke responses different from those observed in this study—a muscular response indicative of fleeing rather than freezing? Would active suppression of empathy or perspective-taking for painful stimuli alter muscular responses? In addition, would suppression effectiveness differ for tasks with more visceral-response vs. representational-response evoking images? Possible experimental avenues for theoretical exploration that might support the implications of this study may exist in research examining individuals with unusual perspective-taking in healthy and pathological conditions—i.e., those with mirror-touch synesthesia (Banissy and Ward, 2007) who seem to experience automatic tactile responses when viewing another person experiencing physical stimuli. It may also be informative to study individuals with potential mirroring dysfunctions (Iacoboni and Dapretto, 2006), such as autistic patients who may engage in less automatic perspective taking (Rizzolatti and Fabbri-Destro, 2010).
